# The first three-dimensional visualization of a thrombus in transit trapped between the leads of a permanent dual-chamber pacemaker: a case report

**DOI:** 10.1186/1752-1947-4-359

**Published:** 2010-11-11

**Authors:** Petra Maagh, Thomas Butz, Andreas Ziegler, Axel Meissner, Magnus W Prull, Hans-J Trappe

**Affiliations:** 1 Department of Cardiology and Angiology, Ruhr-University Bochum/Germany, Hölkeskampring 40, 45625 Herne, Germany; 2 Department of Anaesthesiology, Heart Center Bad Krozingen, Südring 15, 79189 Bad Krozingen, Germany

## Abstract

**Introduction:**

Two-dimensional echocardiography is a useful tool in diagnosing cardiac masses. However, the three-dimensional offline reconstruction technique of transesophageal echocardiography might be superior to two-dimensional transesophageal echocardiography in providing additional information of structural details.

**Case presentation:**

We report the case of a 76-year-old Caucasian man with a permanent dual-chamber pacemaker and a worm-like right-heart thrombus in transit. Two-dimensional transthoracic echocardiography and two-dimensional transesophageal echocardiography showed that it was debatable as to whether "the worm" was originating from the leads. Offline three-dimensional transesophageal echocardiography reconstruction technique proved superior in identifying the cardiac mass as a thrombus trapped between the leads of the pacemaker. The thrombus was successfully dissolved by systemic heparin therapy.

**Conclusions:**

The three-dimensional transesophageal echocardiography is useful and effective in patients with implanted pacemakers or defibrillators when other closely competing imaging modalities are contraindicated, such as magnetic resonance imaging. In patients with pacemakers and trapped thrombus in transit for whom surgical therapy might be a high risk, medical therapy seems to offer a safer and convincing alternative. Whether the management of right-heart thrombi has to be modified due to the presence of pacemaker leads is controversial.

## Introduction

In the context of different imaging modalities, two-dimensional (2D) transesophageal echocardiography (TEE) is a useful tool in diagnosing cardiac masses. It is superior to transthoracic technique in defining the morphology of intracardial structures [1]. By contrast, three-dimensional (3D) TEE might be superior to 2D TEE. Its higher spatial resolution and superior visualization provides additional information about intracardiac anatomy and structural details, such as invasion of underlying cardiac structures and points of attachments.

## Case presentation

A 76-year-old Caucasian man with a 24-year history of myocardial infarction, coronary artery bypass graft and a permanent dual-chamber pacemaker (PM, Guidant INSIGNIA I Ultra^®^) was admitted to our centre with a two-day history of progressive dyspnea. Our exam findings were consistent with right and left heart failure. 2D transthoracic echocardiography (TTE, Siemens Acuson Sequoia 512) with a 2.5 to 3.5 MHz ultrasound transducer revealed an enlarged right ventricle (RV), a systolic pulmonary artery pressure of 46 mmHg calculated by tricuspid regurgitation, and a reduced left ventricular ejection fraction of 20 percent.

**Figure 1:**
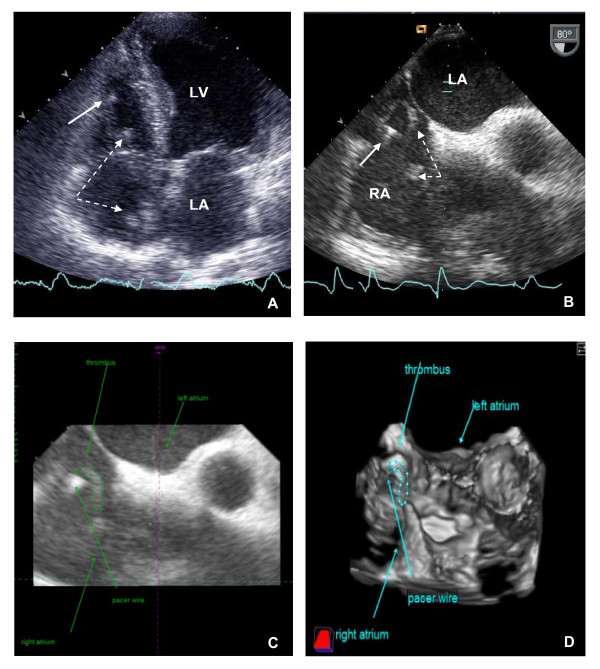
**Echocardiogram showing the thrombus in transit trapped between the permanent pacemaker leads**: [A] four-chamber view of two dimensional transthoracic echocardiography; [B] modified mid-esophageal aortic short-axis view of two dimensional transesophageal echocardiography (80°); [C/D] three-dimensional offline reconstruction of transesophageal echocardiography (80°); [LA] left atrium; [LV] left ventricle; [RA] right atrium; [thick arrow] pace wire; [broken arrow] worm-like thrombus.

Pharmacotherapy with loop diuretics led at first to a symptomatic benefit but was then followed by acute onset of dyspnea with pain in his lower limbs; D-Dimer were elevated to 2222 ng/ml. Our clinical suspicion of a deep venous thrombosis (DVT) was confirmed by venous limb sonography. Pulmonary embolism (PE) was immediately ruled out by computed tomography (CT). The electrocardiogram revealed an atrial flutter with a ventricular rate of 150 per minute; a possible pathomechanism for his symptoms. For the preparation of an external cardioversion, another 2D TTE was performed, demonstrating an echogenic worm-like mass with a length of 10 cm in the right atrium (RA) (see Figure 1A and additional file 1 [Movie 1]); it was also seen in the 2D TEE using a 5-MHz multiplane imaging transducer (see Figure 1B and additional file 2 [Movie 2]). We immediately administered systemic heparin therapy. The atrial flutter converted spontaneously to sinus rhythm.

A surgical thrombectomy was judged to be inappropriate for our patient due to his stable hemodynamic conditions and the high surgical risks posed by his advanced age and his previous cardiac surgery and left ventricular dysfunction. However, due to the poor image quality it remained unclear as to whether the thrombus was trapped between the atrial and ventricular lead of the PM or if it was originating from one of them. 3D visualization of the thrombus demonstrated that the mass was not attached to the RA or RV leads but trapped between them (see Figure 1C, D and additional file 3 [Movie 3]). Three days later, 2D TTE and 3D TEE demonstrated the worm-like formation to be smaller; after one week, 2D TTE and 3D TEE showed a resolution of the right heart (RH) thrombus. Overlapping with the heparin therapy, we initiated oral anticoagulant therapy and continued with an International Normalized Ratio (INR) 2.0 - 3.0. He had an uneventful recovery and follow-up period.

## Discussion

We report the case of a patient with a permanent dual-chamber PM and a worm-like RH thrombus in transit due to a DVT in his lower limb. 2D TTE and TEE showed that it was debatable as to whether "the worm" was originating from the leads. The technology of an offline 3D TEE reconstruction technique helped us to identify that the mass was trapped between the leads of the PM and defined the origin of the thrombus. Although 3D TEE is a time-consuming imaging modality it proved very helpful in this particular case.

The optimal management of RH thromboemboli remains controversial; investigators have recommended either urgent surgical treatment or thrombolysis of mobile RH thrombus, although prospective data for these optimal treatments is lacking [2]. Anticoagulation and thrombolysis are known to reduce the size of the thrombi present in the cardiac and pulmonary vasculature, but they also increase the risk of fragmentation which can lead to further embolization.

In our case report, the thrombus formation was treated successfully with systemic heparin therapy. Our patient was not scheduled for urgent surgical removal of the mass because of his stable hemodynamic conditions and his predicted mortality; calculated by the logistic EuroScore (European System for Cardiac Operative Risk Evaluation [3]) to be at 30.2 percent. Due to the uneventful course, we refrained from performing a lung CT.

The development of RH thrombotic complications in the presence of permanent PM leads has been described in the literature [4]. Serious thrombotic and embolic complications are reported to occur in 0.6 to3.5 percent of patients with permanent transvenous pacing leads [5]. The cases usually describe PM-associated thrombosis [6], and less frequently right atrial PM lead thrombosis [7]. In patients with a mobile RH thrombus, the incidence of pulmonary embolism is 97 percent and reported mortality is over 44 percent [8]. Previously, a giant free-floating right atrial thrombus, comparable with our "worm in the heart", has been described in the literature but the patient died before the initiation of thrombolysis due to fulminant PE [9]. In our case report, the PM leads, instead of generating thrombus, may have acted protectively by trapping the thrombus and may have prevented a fulminant PE. It is remarkable that two patients with PM have been described in the literature to have RH thrombi without fulminant PE. One patient is our own case report. The other report describes an extensive right atrial and ventricle thrombus formation encircling a temporary pacing wire in a patient with heparin-induced thrombocytopenia type II [10]. Thrombolysis leads to complete resolution of all clots documented by TTE and TEE.

## Conclusions

We conclude that 3D TEE seems to be very helpful for the assessment of RH cavities and intracardiac masses in patients with implanted PM or defibrillators. Medical therapy might offer a safe and convincing alternative to surgical therapy in high-risk patients with PM and trapped thrombus in transit. Even though the number of patients with both PM leads and RH thrombus is very small, it is possible that PE is prevented by PM in some patients.

## Consent

Written informed consent was obtained from the patient for publication of this case report and any accompanying images. A copy of the written consent is available for review by the Editor-in-Chief of this journal.

## Competing interests

The authors declare that they have no competing interests.

## Authors' contributions

PM analyzed and interpreted patient data regarding the cardiac disease and was a major contributor in writing the manuscript. PM performed the transthoracic echocardiography. PM, TB and MP performed the transthoracal and transesophageal echocardiography. PM, TB, MP and AZ analyzed the images of the offline reconstruction. AM and HT have been involved in drafting the manuscript and revising it critically for important intellectual content. All authors read and approved the final manuscript.

## Supplementary Material

Additional file 1 **"The worm" presented in two-dimensional transthoracic echocardiography**. Two-dimensional transthoracic echocardiography demonstrated an echogenic worm-like mass with a length of 10 cm in the right atrium trapped between the permanent pacemaker leads and prolapsing through the tricuspid orifice in the right ventricle. Click here for file

Additional file 2 **"The worm" presented in two-dimensional transesophageal echocardiography**. Two-dimensional transesophageal echocardiography from "a worm in the heart". Click here for file

Additional file 3 **"The worm" presented in three-dimensional offline reconstruction of the two-dimensional transesophageal data**. Three-dimensional offline reconstruction of the two-dimensional transesophageal data helped us to identify the origin of the thrombus. 3D visualization of the thrombus demonstrated that the mass was not attached to the leads in the RA and RV but trapped between them. Click here for file
